# Protective Effects of *Phyllanthus amarus* Against Lipopolysaccharide-Induced Neuroinflammation and Cognitive Impairment in Rats

**DOI:** 10.3389/fphar.2019.00632

**Published:** 2019-06-04

**Authors:** Akilandeshwari Alagan, Ibrahim Jantan, Endang Kumolosasi, Satoshi Ogawa, Maizaton Atmadini Abdullah, Norazrina Azmi

**Affiliations:** ^1^Drug and Herbal Research Centre, Faculty of Pharmacy, Universiti Kebangsaan Malaysia, Kuala Lumpur, Malaysia; ^2^School of Pharmacy-SRI, Faculty of Health & Medical Sciences, Taylor’s University, Subang Jaya, Malaysia; ^3^Brain Research Institute, Jeffrey Cheah School of Medicine and Health Sciences, Monash University Malaysia, Bandar Sunway, Malaysia; ^4^Department of Pathology,Faculty of Medicine and Health Sciences, Universiti Putra Malaysia, Serdang, Malaysia

**Keywords:** *Phyllanthus amarus*, neuroinflammation, neuroprotection, non-spatial memory, pro-inflammatory markers

## Abstract

**Background:** *Phyllanthus amarus* (PA) is widely studied for its hepatoprotective properties but has recently received increasing attention due to its diverse anti-inflammatory effects. However, the effects of PA in modulating immune responses in the central nervous system leading to protection against functional changes remain unexplored. Therefore, we sought to examine the protective effects of 80% v/v ethanol extract of PA on lipopolysaccharide (LPS)-induced non-spatial memory impairment and neuroinflammation.

**Methods:** Selected major phytoconstituents of PA extract were identified and quantified using high-performance liquid chromatography. Subchronic neurotoxicity was performed in male Wistar rats given daily oral administration of 100, 200, and 400 mg/kg of the PA extract. Their neurobehavioral activities (functional observation battery and locomotor activity) were scored, and the extracted brains were examined for neuropathological changes. Rats were treated orally with vehicle (5% Tween 20), PA extract (100, 200, and 400 mg/kg), or ibuprofen (IBF; 40 mg/kg) for 14 and 28 days before being subjected to novel object discrimination test. All groups were challenged with LPS (1 mg/kg) given intraperitoneally a day prior to the behavioral tests except for the negative control group. At the end of the behavioral tests, the levels of tumor necrosis factor-α (TNF-α), interleukin (IL)-1β, nitric oxide (NO), inducible nitric oxide synthase (iNOS), CD11b/c integrin expression, and synaptophysin immunoreactivity were determined in the brain tissues.

**Results:** Gallic acid, ellagic acid, corilagin, geraniin, niranthin, phyllanthin, hypophyllanthin, phyltetralin, and isonirtetralin were identified in the PA extract. Subchronic administration of PA extract (100, 200, and 400 mg/kg) showed no abnormalities in neurobehavior and brain histology. PA extract administered at 200 and 400 mg/kg for 14 and 28 days effectively protected the rodents from LPS-induced memory impairment. Similar doses significantly (*p* < 0.05) decreased the release of proteins like TNF-α, IL-1β, and iNOS in the brain tissue. NO levels, CD11b/c integrin expression, and synaptophysin immunoreactivity were also reduced as compared with those in the LPS-challenged group.

**Conclusion:** Pre-treatment with PA extract for 14 and 28 days was comparable with pre-treatment with IBF in prevention of memory impairment and alleviation of neuroinflammatory responses induced by LPS. Further studies are essential to identify the bioactive phytochemicals and the precise underlying mechanisms.

## Introduction

*Phyllanthus amarus* Schumah & Thonn. (PA) belongs to the Euphorbiaceae family and is traditionally used for kidney ailments, diabetes, pain, jaundice, gonorrhea, chronic dysentery, skin ulcer, and hepatitis B. Recently, the plant has received increasing attention and has been studied for various pharmacological properties such as immunomodulatory, antinociceptive, anti-inflammatory, antioxidant, antibacterial, anticancer, antiulcer, gastroprotective, antifungal, antiplasmodic, antiviral, aphrodisiac, contraceptive, hepatoprotective, antihyperglycemic, antilipidemic, nephroprotective, and anti-amnesic activities (Joshi and Parle, 2007; Patel et al., 2011). Although it demonstrates a wide spectrum of pharmacological actions, the unifying features of all these actions are directed towards the anti-inflammatory and antioxidant properties of the plant. PA contains various phytoconstituents such as lignans, alkaloids, phenolics, terpenes, tannins, flavonoids, sterols, and volatile oils (Patel et al., 2011). Of all these phytochemicals, phyllanthin, hypophyllanthin, corilagin, and geraniin are found in abundance and potentially responsible for the reported anti-inflammatory actions of PA (Patel et al., 2011; Jantan et al., 2014). Most of the anti-inflammatory studies were performed in models of inflammation either *in vitro* or *in vivo*. However, limited available data to substantiate the effects of PA in neuroinflammation have warranted a study to explore the anti-inflammatory actions of PA in the central nervous system (CNS).

Neuroinflammation has been implicated in the pathogenesis of neurodegenerative diseases such as Alzheimer’s disease (AD), Parkinson’s disease (PD), and multiple sclerosis (MS) and of neuropsychiatric disorders such as major depressive disorder, schizophrenia, and bipolar disorder (Radtke et al., 2017). Unlike other systemic immune responses, microglia are the resident macrophage in the brain. Microglial activation forms the basis of many immune-mediated responses of the CNS, which enables it to cope with pathogens, toxins, trauma, and degeneration. However, it is the hyperactivation of microglial cells that initiates excessive production of inflammatory mediators leading to destructive inflammatory responses in the brain (Kempuraj et al., 2016). Additionally, the brain is also exposed to constitutive defense responses, such as systemic inflammation. The systemic inflammation may also lead to the initiation of circulating cytokines, thereby impacting CNS and triggering neuroinflammation. Over the decades, it was evident that the immune system plays a central role in learning, memory modulation, and neural plasticity. Under normal quiescent conditions, immune mechanisms positively regulate the neural circuits remodeling, promotion of memory functions, and neurogenesis (Thomson and Sutherland, 2005). In conditions under which the immune system is strongly activated by infection, injury, or severe or chronic stressful conditions, the inflammatory mediators disrupt the delicate balance needed for neurophysiological actions and produces detrimental effects on memory, neural plasticity, and neurogenesis (Okun et al., 2012).

Bacterial infections initiate innate immune responses including activation of toll-like receptor 4 (TLR4) and the transcription factor nuclear factor kappa-light-chain-enhancer of activated B cells (NF-κB) in microglia and macrophages, which subsequently provoke the expression of cytokines and generation of nitric oxide (NO) (Parajuli et al., 2012). These pathways play a basic role in the degradation of bacteria by immune cells, which unfavorably influence neuronal death. Lipopolysaccharide (LPS) is a cell wall component of gram-negative bacteria and a ligand of TLR4 that initiates immune responses to infections. As compared with other glial cells, microglial cells are markedly responsive to LPS leading to learning and memory impairment. Intraperitoneal injection of LPS induces secretion of pro-inflammatory cytokines resulting in neuroinflammation, hippocampal apoptosis, cognitive impairment, learning deficits, and even beta-amyloid plaques generation in the hippocampus (Rock et al., 2004; Zarifkar et al., 2010).

Previous studies in our laboratory identified the presence of phyllanthin, hypophyllanthin, gallic acid, geraniin, corilagin, ellagic acid, and niranthin in 80% ethanol extract of PA (Jantan et al., 2014). It was also found that *P. amarus* at doses of 100 to 500 mg/kg for 14 days revealed non-toxic effect with no abnormalities in general behavior and physiology of rats (Ilangkovan et al., 2015). Additionally, a single or daily repeated doses administration of PA for 28 days revealed no morphological changes in histopathological observation of the kidney, liver, and pancreas (Lawson-Evi et al., 2008; Kushwaha et al., 2013). Lack of study for assessment of neurotoxicity of PA has led us to examine the effects of this plant extract on neurobehavior and brain histopathological changes in rats.

Although the anti-inflammatory activities of PA have been documented (Ilangkovan et al., 2015; Harikrishnan et al., 2018), there is a lack of evidence to substantiate similar effects in the CNS. Treatment with PA extract and phyllanthin was found to improve memory impairment and exhibited anticholinesterase activity in young and older mice (Joshi and Parle, 2006; Joshi and Parle, 2007). These are important early findings that demonstrated the plant activity in the brain suggestive of its potential value in the prevention and treatment of neurodegenerative diseases. Similarly, other *Phyllanthus* species such as *Phyllanthus niruri* (Ambali et al., 2012) and *Phyllanthus emblica* (Ashwlayan and Singh, 2011) have also been reported to reverse memory deficits induced by scopolamine, sodium nitrite, or chlorpyrifos in different animal models of cognitive behavior, which further support a notion of their neuroprotective role. Therefore, the present study sought to examine the neuroprotective effects of PA extract as compared with IBF, a widely studied nonsteroidal anti-inflammatory drug, for its neuroprotective effects against LPS-induced memory impairment and inflammation in rodents.

## Materials and Methods

### Animals

Adult male Wistar rats weighing 190–200 g (5 weeks old) were obtained from the Laboratory Animal Resource Unit (LARU), Universiti Kebangsaan Malaysia (UKM), Malaysia. The rats were housed in a temperature-controlled room (22–25°C) and exposed to 12 h dark/light cycles. Experiments were carried out on the basis of procedures approved by UKM Animal Ethics Committee. Animals were allowed to acclimatize for 7 days before the initiation of treatment. The animal laboratory was maintained under standard conditions. The studies were performed according to procedures for the use of animals in research as approved by the UKM Animal Ethics Committee with the approval number FF/2017/NORAZRINA/24-MAY/850-JUNE-2017-JULY-2018 for the toxicity assessment in rats and FF/2015/NORAZRINA/20-MAY/683-MAY-2015-MAY-2016 for the efficacy and molecular study done in rats.

### Chemicals

LPS from *Escherichia coli* (055:B5) and ibuprofen (IBF; >98% purity) were purchased from Sigma Chemical Co. (St. Louis, MO, USA). Radioimmunoprecipitation assay (RIPA) buffer, enzyme-linked immunosorbent assay (ELISA) kits for tumor necrosis factor-α (TNF-α), and interleukin (IL)-1β were obtained from R&D Technology (Minneapolis, MN, USA). Inducible nitric oxide synthase (iNOS), anti-CD11b/c, and synaptophysin were from Abcam (Cambridge, MA, USA). β-Actin was from Cell Signaling Technology (Beverly, MA, USA). Methanol and acetonitrile [high-performance liquid chromatography (HPLC) grade] were brought from Fisher Scientific (Loughborough, UK). Phyllanthin, hypophyllanthin, gallic acid, geraniin, corilagin, ellagic acid, niranthin, phyltetralin, and isonirtetralin were purchased from ChromaDex (CA, USA) with purity > 98%.

### Preparation of Extract

The whole plant of PA Schumah & Thonn. was obtained from Marang, Kuala Terengganu, Malaysia, in the month of February 2016. Dr. Abdul Latif Mohamad from the Faculty of Science and Technology, Universiti Kebangsaan Malaysia (UKM), identified the plant, and a voucher specimen (*P. amarus* UKMB 30078) was deposited at the Herbarium of UKM, Bangi, Malaysia. After collection, the plant was allowed to dry for a week and then ground to form the coarse powder. A total of 1 kg of the coarse powder was soaked in 80% ethanol for 9 days. The extract was filtered, and the solvent was changed every 72 h. The solvent was removed using a rotary evaporator, and the extract was subjected to freeze-drying and stored in −20°C.

### High-Performance Liquid Chromatography Analysis of 80% Ethanol Extract of *Phyllanthus amarus*

Qualitative and quantitative HPLC analysis was performed according to the method of Jantan and colleagues, with a slight modification (Jantan et al., 2014). Twenty milligrams of PA extract and 1 mg of reference standards (gallic acid, ellagic acid, corilagin, geraniin, niranthin, phyllanthin, hypophyllanthin, phyltetralin, and isonirtetralin) each in 1 mL of methanol were ultra-sonicated for 10 min. Then they were filtered through 0.45-µm Millipore Millex polytetrafluoroethylene membrane (Maidstone, Kent, UK). The diluted extract and the reference standards were analyzed using the following conditions: reversed-phase column, C-18 (100 mm × 4.6  mm inner diameter, 5 µm, XBridge, Waters, Ireland); detector, photodiode array (Waters 2998); wavelength, 220 nm; and flow rate, 1.0 mL/min. Phyllanthin and hypophyllanthin were eluted isocratically (mobile phase A, acetonitrile; B, acidified water with 0.1% orthophosphoric acid) with 5% B, increased to 95% over 20 min, and then followed by 95% for 15 min. In addition to that, gallic acid, ellagic acid, corilagin, geraniin, niranthin, phyltetralin, and isonirtetralin were eluted by gradient method (mobile phase A, acetonitrile; B, acidified water with 0.2% orthophosphoric acid) with 5–70% A (0 to 15 min) and 70% to 95% A (15 to 30 min) and then followed by 95% A for 20 min. Compound identification was done by comparing the retention time and spectra peaks with the reference standards. A calibration curve was plotted with three different concentrations (250, 500, and 1,000 µg/mL) for each standard versus the area under the peaks. The standard curve equations obtained from each bioactive compound were used to quantify the compounds in the extract (Jantan et al., 2014; Yuandani et al., 2016).

### High-Performance Liquid Chromatography Validation

Validation was performed by determining the linearity, precision, limit of detection (LOD), and limit of quantification (LOQ). Linear calibration analysis was evaluated to determine the linearity by calculating the correlation coefficient (*r* ^2^) from the calibration curve. A calibration curve was plotted with three different concentrations (250, 500, and 1,000 µg/mL) for each standard. The method precision was determined by intra-day and inter-day assays, and they are performed by injecting the extract (20 mg/mL) and standards (1 mg/mL) separately three times in a day and on three different days. The mean values of the area under the peak and retention time obtained from the intra-day and inter-day assay were used to confirm the reproducibility of the results. LOD and LOQ were calculated using the formula LOD = 3.3 × (σ/*S*) and LOQ = 10 × (σ/*S*), where σ = standard deviation of the response and *S* = slope.

### Subchronic Neurotoxicity Study

Toxicity tests were conducted according to Organisation for Economic Co-operation and Development 423 guidelines with modified procedure (Organisation for Economic Co-operation and Development, 2002). Twenty adult male Wistar rats were divided into four groups randomly. Group 1 was considered as vehicle control with 5% Tween 20; groups 2, 3, and 4 were administered orally with 100, 200, and 400 mg/kg of PA extract for 28 days. The general behaviors of rats were observed by functional observational battery (FOB) test and followed by histopathological evaluation.

#### Functional Observational Battery

Neurobehavioral changes were evaluated by FOB, a widely used screening method to identify potential neurotoxicity of new and existing chemicals. The procedure was based on the McDaniel and Moser (1993) protocol with slight modifications. Home-cage movement and hand-held observations were performed in the FOB. The number of line crossings, rearing against a wall, center square entry, grooming, defecation, and urination were observed in the open field activity test for 28 days (McDaniel and Moser, 1993; Brown et al., 1999).

#### Histopathological Evaluation

After behavioral analysis, rats were sacrificed, and their brains were dissected and weighed. They were immersed in 10% neutral buffered formalin and stored until further analysis. Tissues were processed, embedded in paraffin, and sectioned at 5-μm thickness. The slides were stained with hematoxylin and eosin (H&E) and viewed under a light microscope for semiquantitative histological evaluation (Clausen et al., 2009) and morphological observation of the neurons and pyramidal cells. Responses to insult were evaluated as edema of the brain parenchyma and infiltration of the cortical lesion and hippocampus ipsilaterally by macrophages, neutrophils, and lymphocytes as the signs of inflammation. Evaluation of morphological and pathological changes was performed by an investigator (MAM) blinded to the treatment status of each animal.

### Experimental Design for 14 and 28 Days of *Phyllanthus amarus* Extract Treatment

The animals were divided into six groups of eight and received daily oral administration of 5% Tween 20 (negative and vehicle control groups), IBF 40 mg/kg (positive control), and PA extract at different doses (100, 200, and 400 mg/kg). The treatment was scheduled for 14 and 28 days. A day prior to the cognitive behavioral tests, all groups were challenged with 1 mg/kg of LPS given intraperitoneally except the negative control group, which received an intraperitoneal injection of the vehicle. The animals were decapitated at the end of the experiment, and their brains were collected for further analysis.

### Cognitive Behavioral Studies

#### Novel Object Discrimination Task

Novel object discrimination test (NOD) is utilized to assess the non-spatial memory in rodents. This test examines the time spent in exploring the novel object and a familiar object. The method was adopted from previous studies with minor modifications (Ennaceur, 2010; Azmi et al., 2011). NOD was performed in an open box (width × length × height = 40 cm × 40 cm × 40 cm) made of Perspex. The lighting setup was fixed above the ceiling inside the behavior room with constant illumination. The object was a glass bottle made with a distinct color. The floor was divided into nine equal squares by black lines for scoring of locomotor activity. The procedure consisted of three phases: habituation, familiarization, and test phases. Each rat was pre-habituated for 1 h per day before the experiment. On the experiment day, animals were allowed to explore the empty arena for 3 min individually, followed by the familiarization phase, where an animal was allowed to explore two identical objects (A1 and A2) for another 3 min. During the test phase, animals were allowed to explore one identical object that has been explored before (A3) and one novel object (B) inside the arena for 3 min. In between phases, the open field and objects were cleaned with 70% v/v ethanol to eliminate olfactory cues while the rat was returned to its home cage. The location and combination of objects used were alternated between rats to decrease bias. Scoring of exploration was carried out when the rat nose was directed towards the object at a distance of ≤2 cm or touching the object. Turning around, sitting, or climbing on the object was not scored as an exploratory behavior. Their exploratory behavior was scored during a playback of the closed-circuit television (CCTV) footage. The non-spatial memory was determined using the values of discrimination index (DI). DI was calculated for every rat using the formula DI = (B − A3)/(B + A3).

#### Locomotor Activity

The locomotor activity was measured for each rat by scoring the number of line crossings inside the arena during familiarization and test phases. The activity was scored as the frequency of line crossings with all four paws across the line during a playback of the CCTV footage.

### Brain Sample Collection

After the behavioral study, animals were decapitated and their brains dissected into two hemispheres to be stored in −80°C until further analysis. The left and right hemispheres were collected alternately from each group and homogenized in an equal amount of RIPA (R&D Technology, Minneapolis, MN, USA) buffer with protease inhibitor followed by centrifugation at 12,000 rpm for 20 min at 4°C. The supernatant was collected and molecular analysis performed.

### Measurement of Cytokines

The concentrations of pro-inflammatory cytokines TNF-α and IL-1β were estimated using ELISA kits (R&D Systems, Minneapolis, MN, USA) as per manufacturer protocol. Cytokine levels were expressed as percentage of inhibition (Achoui et al., 2010; Harikrishnan et al., 2018). The percentage inhibition was calculated as

$$\%\;Inhibition=100\times \left\{\frac{\left[{\left(cytokine\right)}_{control}-{\left(cytokine\right)}_{sample}\right]}{{\left(cytokine\right)}_{control}}\right\}$$

### Griess Assay

Nitrite levels were measured in the brain tissue using the Griess assay. The Griess reagent consisted of 0.1% *N*-(1-naphthyl)ethylenediamine dihydrochloride, 1% sulfanilamide, and 2.5% phosphoric acid. Equal volume of Griess reagent and brain sample was mixed and incubated at 37°C for 10 min. The absorbance was read at 542-nm wavelength spectrophotometrically in ELISA plate reader. Nitrite levels were expressed as percentage of inhibition (Green et al., 1982; Achoui et al., 2010). The percentage inhibition was calculated as

$$\%\;Inhibition=100\times \left\{\frac{\left[{\left(NO_{2}\right)}_{control}-{\left(NO_{2}\right)}_{sample}\right]}{{\left(NO_{2}\right)}_{control}}\right\}$$

### Western Blot

Total protein concentrations in the left and right hemispheres were quantified using NanoQuant. Equal amount of protein (60 µg) was separated by 8% and 12% sodium dodecyl sulfate polyacrylamide gel electrophoresis and transferred to polyvinylidene fluoride membrane. Blots were blocked for 2 h at room temperature with 1% bovine serum albumin (BSA) in tris-buffered saline with Tween 20 (TBST) and washed. The blots were incubated with primary antibody iNOS (1:1,000, Abcam, Cambridge, MA, USA) and synaptophysin (1:500, Abcam, Cambridge, MA, USA) overnight. After being washed, blots were incubated with horseradish peroxidase-conjugated secondary antibody for 2 h. The membrane was then developed for visualizing the protein band in gel doc image analyzer using enhanced chemiluminescence kit (R&D Systems) (Zhu et al., 2016; Harikrishnan et al., 2018).

### Immunohistochemistry of Brain Slices

After the behavioral study, two rats from each group were anesthetized with a combination of ketamine, xylazine, tiletamine, and zolazepam. Transcardiac perfusion with normal saline (0.9%) and buffered 4% paraformaldehyde was performed to fix the brain. The whole brain was collected and cryoprotected with 30% sucrose. The hippocampus area was sectioned coronally in the cryostat (30 µm in thickness) and stored in anti-freezing solution. Then the sections were washed twice with 0.1M phosphate-buffered saline (PBS). The sections were blocked with 3% BSA, 0.75% Triton X, and 0.1M PBS for 1 h in 60 rpm at room temperature. They were incubated overnight with mouse monoclonal anti-CD11b/c (1:500, Abcam, Cambridge, MA, USA). The next day, the sections were washed twice and kept for 1-h incubation with Alexa Fluor 488 secondary antibody. Finally, the sections were mounted on slides after being washed and viewed under a confocal microscope (Nikon Microscope ECLIPSE TE 2000-E) with a 20× objective. The immunostained area in the hippocampus for CD11b/c was measured using ImageJ software referred to a particular area (4 × 10^4^ μm^2^) (Hernangomez et al., 2016; Shen et al., 2016).

### Statistical Analysis

Data obtained were analyzed by one-way analysis of variance (ANOVA) followed by Dunnett’s *post hoc* test using GraphPad Prism 5. All experimental data were expressed as mean ± standard error of mean (SEM), where “*n*” denotes the number of rats involved in the test. *p* < 0.05 value was set as statistically significant.

## Results

### Qualitative and Quantitative Analysis of 80% Ethanolic Extract of *Phyllanthus amarus*

**Figure 1:**
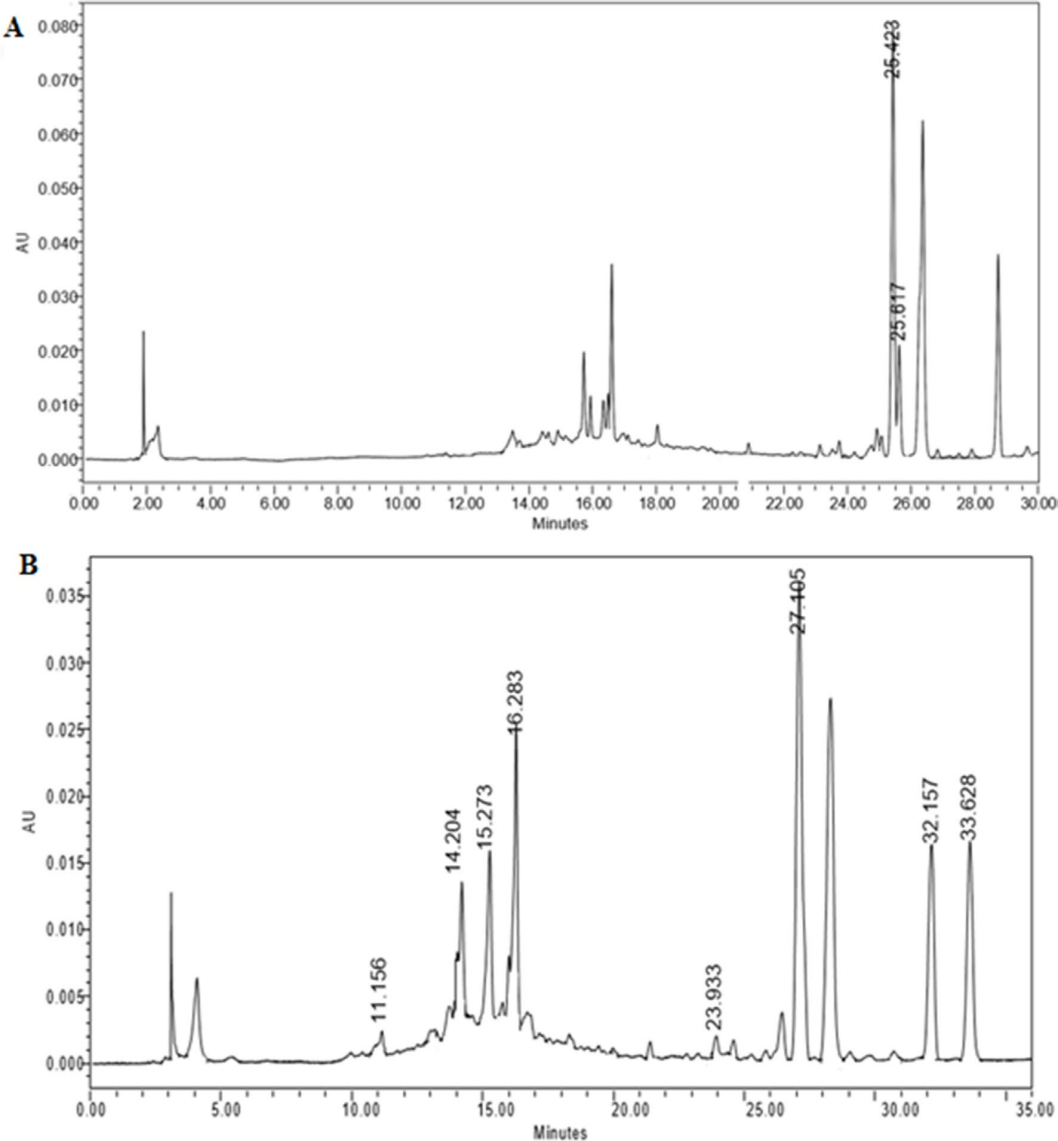
**(A)** High-performance liquid chromatography (HPLC) chromatogram profiling of *Phyllanthus amarus* (PA) extract with identified retention time of phyllanthin (25.423 min) and hypophyllanthin (25.617 min). **(B)** Retention time of gallic acid (11.156 min), geraniin (14.204 min), corilagin (15.273 min), ellagic acid (16.283 min), niranthin (23.933 min), phyltetralin (32.157 min), and isonirtetralin (33.628 min) in the extract.

Identification of major phytochemicals in 80% ethanol extract of PA was carried out using HPLC. Separation of phyllanthin and hypophyllanthin was identified at 25.423 and 25.617 min, respectively (**Figure 1A**). Gallic acid, geraniin, corilagin, ellagic acid, niranthin, phyltetralin, and isonirtetralin appeared at 11.156, 14.204, 15.273, 16.283, 23.933, 32.157, and 33.628 min, respectively (**Figure 1B**). Compound identification was accomplished by comparing retention time with respective standard compounds (**Table 1**). The LOD and LOQ of the identified compounds are listed in **Table 1**. Among the identified compounds in PA extract, ellagic acid (218.06 µg/mL) content was found to be the highest followed by phyllanthin (162.69 µg/mL), corilagin (158.68 µg/mL), phyltetralin (145.83 µg/mL), niranthin (102.97 µg/mL), hypophyllanthin (95.37 µg/mL), isonirtetralin (71.80 µg/mL), geraniin (67.47 µg/mL), and gallic acid (18.32 µg/mL).

**Table 1:** Limit of detection (LOD) and limit of quantification (LOQ) of the identified compounds.

Standards	RT	Intra-day (*n* = 3)		Inter-day (*n* = 3)	
		RT (RSD) (%)	Peak area (RSD) (%)	RT (RSD) (%)	Peak area (RSD) (%)
Gallic acid	11.15	1.297	6.638	1.014	4.742
Geraniin	14.20	0.500	3.501	0.285	4.096
Corilagin	15.27	0.277	1.083	0.294	1.066
Ellagic acid	16.28	2.149	4.507	0.421	4.715
Niranthin	23.93	0.216	0.303	0.164	7.567
Phyllanthin	25.42	0.087	3.792	0.017	5.364
Hypophyllanthin	25.61	0.146	2.104	0.056	4.964
Phyltetralin	32.15	0.714	6.380	0.627	8.230
Isonirtetralin	33.62	0.714	6.380	0.017	7606

RT, retention time; RSD, relative standard deviation.

### Functional Observational Battery

Rats treated with PA extract for 28 days did not show any abnormal behavioral changes while handling and home-cage movement. No significant changes were observed in body weight, and there was no mortality rate. In locomotor activity, PA extract administered rats did not show any significant changes in the frequency of line crossing, rearing, central square entry, defecation, and urination (**Supplementary Figure S1**).

### Histopathological Evaluation of *Phyllanthus amarus*-Treated Brain Tissues

The cerebral cortex and hippocampus were sectioned and stained to identify whether PA extract has shown any morphological changes in rats. The vehicle control group and PA extract-treated group at three different doses did not show any edema or inflammatory changes in the cerebral cortex and hippocampus. In addition, no neuronal morphological changes were observed in the neurons and pyramidal cells (**Supplementary Figure S2**).

### Effects of *Phyllanthus amarus* Extract on Cognitive Behavioral Studies

**Figure 2:**
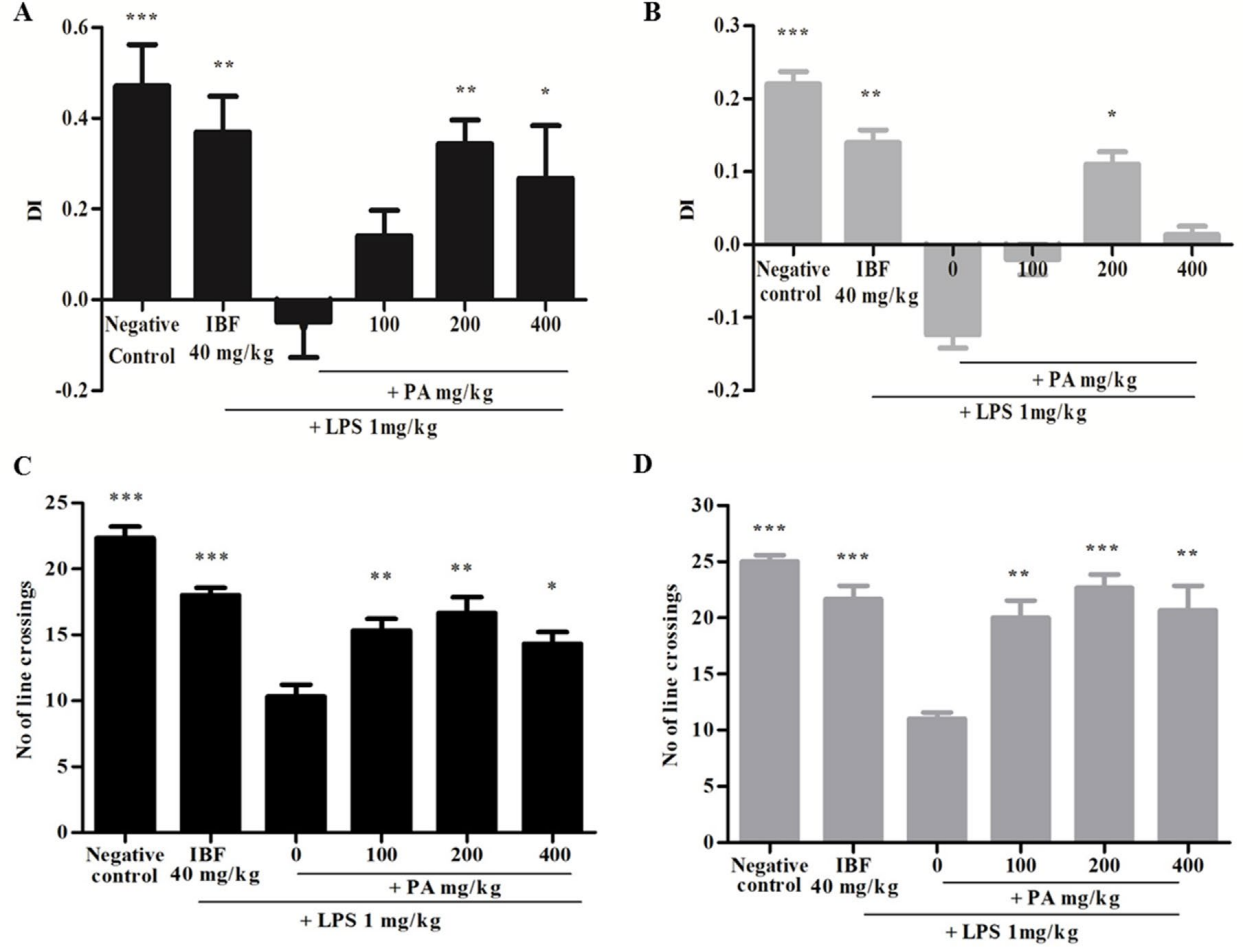
Discrimination index of PA extract treatment after 14 **(A)** and 28 **(B)** days. Data expressed as mean time in seconds ± standard error of the mean [*p < 0.05, **p < 0.01, ***p < 0.001 vs. lipopolysaccharide (LPS), respectively]. Locomotor activity after 14 **(C)** and 28 **(D)** days’ treatment of PA extract. Data expressed as the mean number of line crossings ± standard error of the mean (*p < 0.05, **p < 0.01, ***p < 0.001 vs. LPS, respectively). LPS, lipopolysaccharide; IBF, ibuprofen (positive control); and PA, *Phyllanthus amarus* extract.

The DI was calculated as mentioned earlier and shown in **Figure 2**. **Figure 2A,B** reveals that the group that received LPS alone showed negative DI values, indicating non-spatial memory impairment where the rats spent more time exploring the familiar object than the novel object. Pre-treatment of PA extract (100, 200, and 400 mg/kg) for 14 days demonstrated positive DI, indicating that the rats were able to discriminate the novel from familiar objects significantly (*p < 0.05, **p < 0.01). However, after 28 days of treatment, only groups treated with 200 (*p < 0.05) and 400 mg/kg of PA extract showed a positive DI. Taken together, it was evident that PA extract at 200 and 400 mg/kg protected against LPS-induced memory impairment. Similarly, the positive control (**p < 0.01) group also demonstrated a positive DI value. Locomotor activity was evaluated by the number of line crossings in the test arena. **Figure 2C,D** indicates that all groups demonstrated significantly higher locomotor activity (***p < 0.001, **p < 0.01) than did the group that received LPS alone.

### Effects of *Phyllanthus amarus* Extract on Lipopolysaccharide-Induced Pro-Inflammatory Cytokines

The concentrations of TNF-α and IL-1β proteins were measured in rat brains. Elevation of pro-inflammatory cytokines in the LPS-induced group was observed as an evidence for neuroinflammation through systemic inflammation. PA extract-treated groups showed a significant decrease of TNF-α (***p < 0.001, **p < 0.01, respectively) and IL-1β (*p < 0.05, **p < 0.01, ***p < 0.001, respectively) levels, than did vehicle control in 14- and 28-day treatments. The percentage of inhibition of TNF-α and IL-1β is shown in **Table 2**.

**Table 2:** Percentage inhibition of necrosis factor-α (TNF-α) and interleukin (IL)-1β production.

Treatment	Percentage of inhibition (%)			
	14 days’ treatment		28 days’ treatment	
	TNF-α	IL-1β	TNF-α	IL-1β
Negative control	100***	100***	100***	100***
IBF 40 mg/kg + LPS	78.82 ± 0.43**	76.92 ± 0.51***	78.19 ± 0.57***	69.25 ± 0.58**
Vehicle control (LPS)	0 ± 0.43	0 ± 0.51	0 ± 0.67	0 ± 0.61
PA 100 mg/kg + LPS	77.11 ± 0.52**	72.1 ± 0.74***	69.53 ± 0.81**	55.68 ± 0.46*
PA 200 mg/kg + LPS	87.15 ± 0.32***	79.51 ± 0.43***	80.09 ± 0.72***	75.80 ± 0.61**
PA 400 mg/kg + LPS	81.96 ± 0.63**	75.82 ± 0.41***	74.3 ± 0.71***	72.66 ± 0.52**

TNF-*α*, tumor necrosis factor-*α*; IL-1*β*, interleukin-1-beta; IBF, ibuprofen; PA, Phyllanthus amarus; LPS, lipopolysaccharide. Data expressed as mean ± standard error of the mean (n = 3); ***p < 0.001 and **p < 0.01 compared with vehicle control (vehicle + LPS).

### Determination of Nitric Oxide Levels

Administration of PA extract for 14 and 28 days at three different doses showed significant reduction of NO production (**p < 0.01, ***p < 0.001). NO level in the LPS-induced group was greatly increased than in other groups (**Table 3**).

**Table 3:** Percentage inhibition of nitric oxide (NO) production.

Treatment	Percentage inhibition of NO production (%)	
	14 days’ treatment	28 days’ treatment
Negative control	100***	100***
IBF 40 mg/kg + LPS	69.89 ± 0.15**	68.33 ± 0.22***
Vehicle control (LPS)	0 ± 0.423	0 ± 1.37
PA 100 mg/kg + LPS	67.01 ± 0.11**	59.27 ± 0.08**
PA 200 mg/kg + LPS	74.11 ± 0.07***	73.32 ± 1.55***
PA 400 mg/kg + LPS	67.8 ± 0.31**	64.19 ± 0.66***

TNF-*α*, tumor necrosis factor-*α*; IL-1*β*, interleukin-1-beta; IBF, ibuprofen; PA, Phyllanthus amarus; LP, lipopolysaccharide. Data expressed as mean ± standard error of the mean (n = 3); ***p < 0.001 and **p < 0.01 compared with vehicle control (vehicle + LPS).

### Effects of *Phyllanthus amarus* Extract in Lipopolysaccharide-Induced Changes in Pro-Inflammatory Enzymes and Synaptic Marker

**Figure 3:**
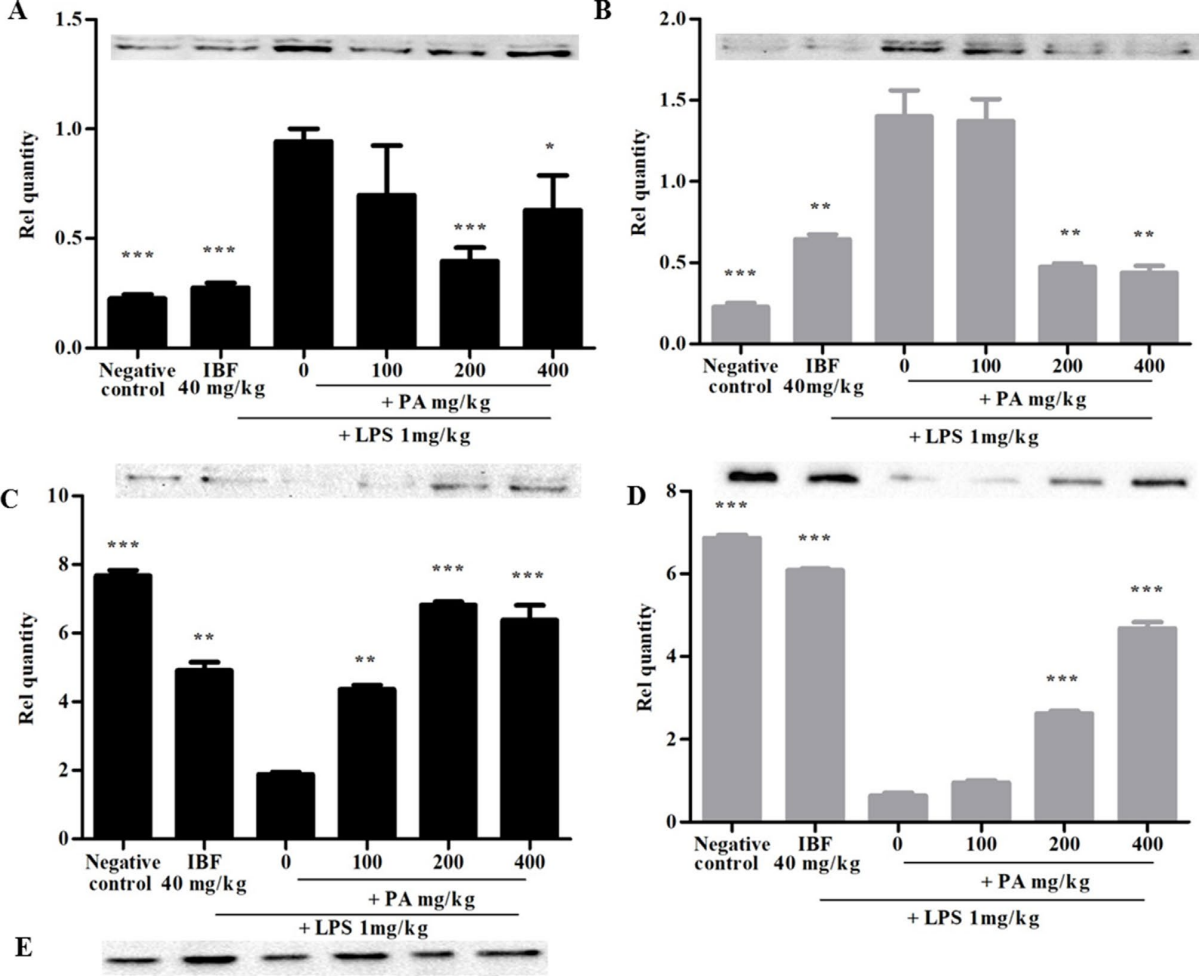
Expression of inducible nitric oxide synthase (iNOS) **(A, B)** and synaptophysin **(C, D)** after 14 **(A, C)** and 28 days’ **(B, D)** treatment of PA extract. **E** refers to β-actin (control used for **A**, **B**, **C**, and **D**). Data expressed as mean ± standard error of the mean (*p < 0.05, **p < 0.01, ***p < 0.001 vs. LPS, respectively). LPS, lipopolysaccharide; IBF, ibuprofen; and PA, *Phyllanthus amarus* extract.

**Figure 3** shows the expression of iNOS protein (A and B) and synaptophysin immunoreactivity (C and D) following 14 and 28 days’ treatment of PA extract in the brain tissue homogenate. The doses of 100 and 200 mg/kg of PA extract significantly decreased the LPS-induced iNOS protein expression (*p < 0.05, **p < 0.01, ***p < 0.001), respectively, after 14 and 28 days of pre-treatment. The levels of presynaptic marker synaptophysin were significantly decreased by LPS (***p < 0.001), whereas in the PA-treated groups, the level of synaptophysin significantly increased (***p < 0.001).

### Protective Effects of *Phyllanthus amarus* Extract Against Glial Cell Activation

**Figure 4:**
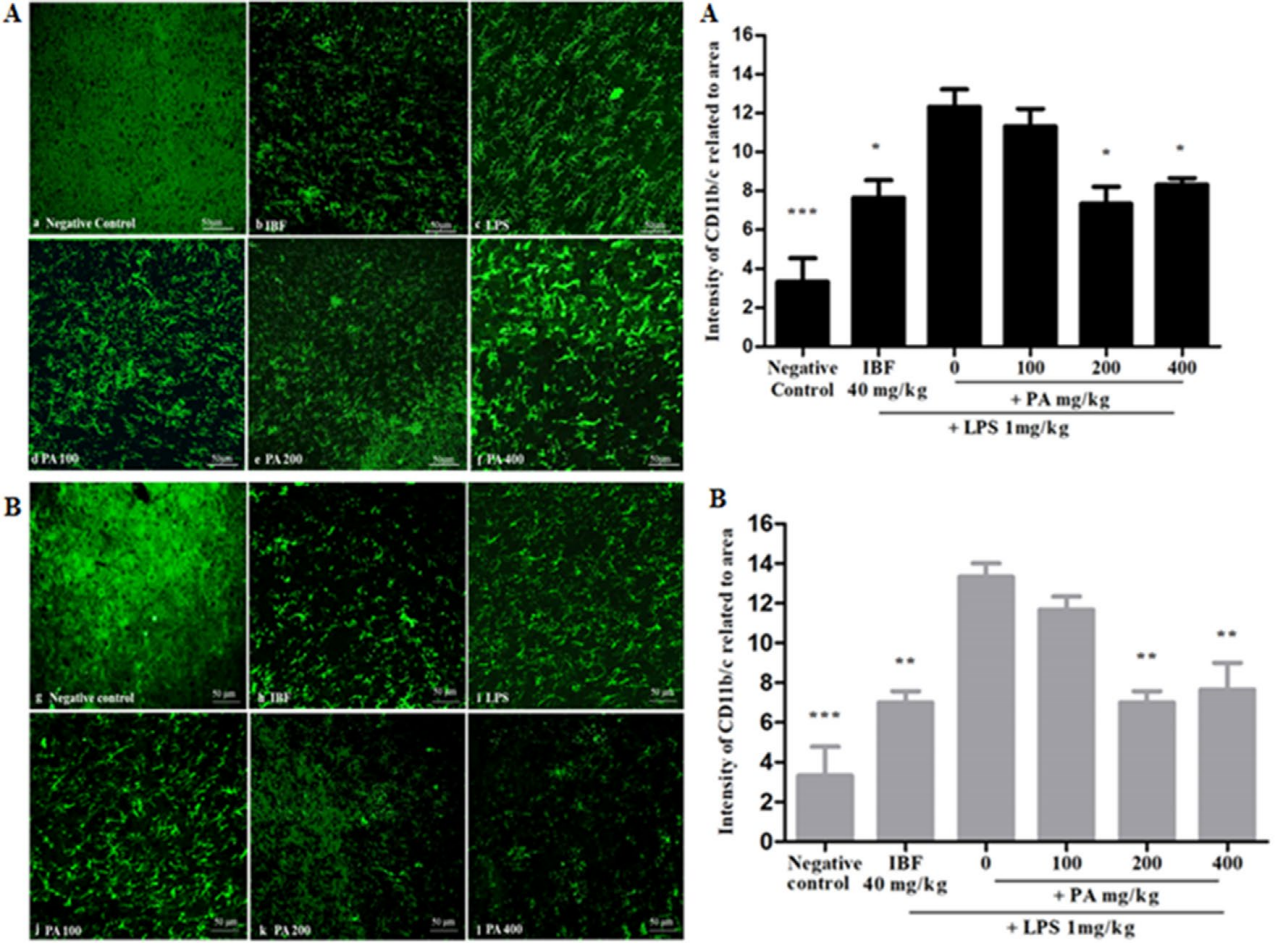
Immunohistochemistry staining of CD11b/c in the hippocampal region of the rat brain (20×): the photomicrographs of brain section presenting the microglial activation in hippocampus after 14 **(A)** and 28 **(B)** days’ treatment. Graphical representation **(A, B)** refers to CD11b/c immunoreactivity area in rats’ hippocampus. Data expressed as mean ± standard error of the mean (*p < 0.05, **p < 0.01, ***p < 0.001 vs. LPS, respectively). LPS, lipopolysaccharide; IBF, ibuprofen (positive control); and PA, *Phyllanthus amarus* extract.

The CD11b/c integrin is expressed in activated microglial as a surface marker. It is an effective marker to recognize microglial activation at the time of neurodegeneration. **Figure 4A,B** shows the immunohistochemistry staining and intensity of CD11b/c integrin expression in the hippocampal region of the rat brain after 14 and 28 days’ administration of PA extract. The LPS-treated group, as compared with other treated groups, showed small and numerous slender cell morphological features, which clearly explains the presence of activated microglia. Pre-treatment of PA extract of 200 and 400 mg/kg in 14 (*p < 0.05) and 28 days (**p < 0.01) showed a significant reduction of CD11b/c integrin expression comparable with that of the positive control.

## Discussion

PA has been widely studied for its anti-inflammatory activity in *in vitro* and *in vivo* models. A previous study demonstrated that PA prevented memory impairment and possessed anticholinergic and anti-inflammatory properties (Joshi and Parle, 2006). These findings suggested that PA is neuroactive and can alter the brain functions. However, to the best of our knowledge, no studies have been done to determine the effects of PA on neuroinflammation. Therefore, the present study sought to investigate the protective effects of PA extract against memory impairment and immune responses in LPS-induced neuroinflammation in rodents. The biological activities of medicinal plants are usually due to the presence of bioactive phytoconstituents. Similar to other studies, our results showed the presence of major phytoconstituents such as gallic acid, geraniin, corilagin, ellagic acid, niranthin, phyllanthin, hypophyllanthin, phyltetralin, and isonirtetralin in PA. The present finding is in accordance with previous studies reported for the presence of lignans and polyphenols in this species (Jantan et al., 2014). We have also demonstrated that subchronic treatment of PA extract did not show any signs of toxicity in the animal behavior. In addition, no pathological changes were detected in pyramidal cells of the hippocampus and cerebral cortex. A similar study in our laboratory showed that 80% ethanol extract of PA at doses ranging from 100 to 500 mg/kg given orally did not cause any abnormal behavior and mortality in rats (Ilangkovan et al., 2015). Taken together, it indicates that the concentrations of the extract used for this study were within the safe limit.

Novel object discrimination task was performed to assess the rodents’ working memory and preferences towards novelty (Ennaceur, 2010). After 14 days’ administration of PA extract (100, 200, and 400 mg/kg), rats showed positive preference towards the novel object than did the vehicle control group. However, 28 days’ administration of PA extract at 200 mg/kg showed a significant positive preference for the novel object but not with the other doses. It is suggested that pre-treatment with PA protected against LPS-induced impairment in non-spatial memory at an optimum dose of 200 mg/kg and prevented hypolocomotion induced by LPS (Custódio et al., 2013; Tortorelli et al., 2015), which were comparable with the effects of IBF. Cognitive studies done with other *Phyllanthus* species, namely, *P. emblica*, *P. niruri*, and *P. reticulates* have also demonstrated reversal of memory impairment possibly due to the anti-cholinesterase activity of this genus (Ambali et al., 2012; Malve et al., 2014; Uddin et al., 2016).

Cognitive impairment is noticeable in Alzheimer’s disease (AD) and also in other disorders where neuroinflammation is proposed to play a prominent role (Ownby, 2010). Initiation of TLR4, which is expressed in astrocytes, microglia, and neuron can antagonistically influence neuronal plasticity and survival in brain injury (Okun et al., 2012). Additionally, TLR4 signaling reduces neurogenesis and results in cognitive impairment. As a consequence, microglial cells are activated and release inflammatory mediators like TNF-α, IL-1β, IL-6, iNOS, NO, reactive nitrogen species, and reactive oxygen species which mediates oxidative stress (Zhang et al., 2018). Indeed, an inflammatory response is a common observation in the brain tissue of patients with dementia (Zhao et al., 2018). Likewise, our present study showed that LPS increased the levels of pro-inflammatory cytokines (TNF-α and IL-1β), which act as a central part in the onset and maintenance of inflammation. Related proteins such as iNOS and NO were significantly increased, and a decrease in the level of synaptic marker was noted after exposure to LPS. Indeed, it is known that high concentrations of NO but not the lower concentrations may advance excitotoxicity and result in cognitive impairment. It was suggested that LPS might stimulate the inflammatory cytokines levels in the brain *via* TLR4 activation and induces neuroinflammation (Zhang et al., 2018).

The immune-mediated changes seen with LPS were effectively prevented by pre-treatment of PA extract at 200 and 400 mg/kg for 14 and 28 days. In fact, previous studies have reported that corilagin from PA, ellagic acid, and geraniin were found to decrease the release of TNF-α and IL-β in the brain (Farbood et al., 2015; Tong et al., 2016). Additionally, there is a correlation between pro-inflammatory cytokine release with the decreased level of synaptophysin, which affects memory status (Strużynska et al., 2006). In the present study, we demonstrated that 200 and 400 mg/kg of PA extract given for 14 and 28 days attenuated the LPS-induced pro-inflammatory cytokine release with resulting synaptic loss and memory impairment suggestive of a protective effect against LPS-induced neuroinflammation. Neuroinflammatory conditions, for example, traumatic brain damage, AD, Down syndrome, and aging are often presented with memory impairment. A relationship between cytokine expression in the brain and memory deficits has also been established. For example, it has been demonstrated that the release of TNF-α and IL-1β is a sign of neuroinflammatory-induced memory impairment. Indeed, acute inflammation induced by LPS or IL-1β infusion led to memory impairment (Shaftel et al., 2007). Similarly, previous findings demonstrated that hippocampal IL-1β overexpression hinders contextual and spatial long-term memory (Thomson and Sutherland, 2005). It becomes increasingly evident that prevention of memory impairment observed in the PA-treated groups could be related to immunological changes in the brain.

Astrocytes, microglia, and neurons respond to the acute or chronic stimulation and can aggressively influence neuronal plasticity and surface marker (Okun et al., 2012) resulting in neuroinflammation. CD11b/c is considered as an active integral marker of the innate immune response in microglial cells. Researches revealed that activation of microglia is often noted by expressing the surface marker CD11b/c in either the administration of endotoxins (for example LPS) or injury (Liu et al., 2016). It is believed that microglial activation expresses various proteins and surface markers. Among them, CD11b/c is more sensitive and an intense marker, which plays a role as a binding protein for intracellular adhesion molecule-1 and complement receptor type 3. Therefore, CD11b/c was examined in the hippocampal region of the brain tissue. The outcome of the present study showed that PA extract and IBF attenuated CD11b/c integrin expression in LPS-induced rat brain, which points to a notion that PA inhibits microglial activation similar to IBF. Studies have shown that increased microglial marker CD11b/c are influenced by NO production. However, their complete signaling mechanism is uncertain to researchers (Nillert et al., 2017).

Most of the changes in cognitive function and immunological markers were optimum at 200 mg/kg of PA extract, and increasing the dose by two-fold did not cause any further increase in the effects. It is highly unlikely that the lack of dose–response effects between 200 and 400 mg/kg in many of the parameters measured was due to toxicity. This is evident from our toxicity study that did not demonstrate any neurotoxicity at the highest dose of 400 mg/kg. The lack of dose–response effects could also be due to saturation of receptors at higher concentrations where a similar observation has been reported in other studies (Lima et al., 2016; Manalo et al., 2017). In the present study, it is unclear as to whether the observed effects were produced by the major phytoconstituents of the plant or as a result of a combined effects of various phytochemicals present in the extract. Indeed, this study sought to determine the efficacy of PA crude extract in modulating inflammatory responses in the brain by measuring only selected markers of neuroinflammation. Although limited, the present findings provide an early indication of the protective actions of PA against non-spatial memory impairment, which may result from inflammatory processes in the brain. It is also unclear if PA alleviated inflammation in the brain by inhibiting TLR4 activation induced by LPS. A different group from our laboratory has similarly demonstrated an anti-inflammatory action of PA through inhibition of LPS-induced responses in human macrophages (Harikrishnan et al., 2018). The study revealed that PA targeted the NF-κB, mitogen-activated protein kinase, and phosphatidylinositol 3-kinase/protein kinase B signaling pathways to exert its anti-inflammatory effects. Therefore, on the basis of our present findings, we suggest that PA exhibited anti-inflammatory actions *via* LPS-induced signaling pathway.

## Conclusion

Pre-treatment with PA extract for 14 and 28 days was comparable with that of IBF in the prevention of non-spatial memory impairment and alleviation of neuroinflammatory responses induced by LPS. However, further investigations are warranted to support this notion and to better understand the exact protective mechanisms of PA in altering immune and inflammatory responses in the brain. Identifying the bioactive compounds in the plant that are responsible for these effects is also essential in future studies.

## Ethics Statement

The studies were performed according to procedures for the use of animals in research as approved by the UKM Animal Ethics Committee with the approval number FF/2017/NORAZRINA/24-MAY/850-JUNE-2017-JULY-2018 for the toxicity assessment in rats and FF/2015/NORAZRINA/20-MAY/683-MAY-2015-MAY-2016 for the efficacy and molecular study done in rats.

## Author Contributions

NA, IJ, and EK were involved in the study design, analysis, and interpretation of the data. AA performed the experiments and data analysis. SO participated in the immunohistochemistry work, and MA analyzed the brain histology. The manuscript draft was written by AA and finalized by NA, IJ, EK, SO, and MA.

## Funding

This study was supported by the Ministry of Agriculture & Agro-Based Industry, Malaysia under the NKEA Research Grant Scheme (NRGS) with grant no. NH1014D023.

## Conflict of Interest Statement

The authors declare that the research was conducted in the absence of any commercial or financial relationships that could be construed as a potential conflict of interest.

## Abbreviations

PA, *Phyllanthus amarus*; LPS, lipopolysaccharide; IBF, ibuprofen; i.p., intraperitoneal injection; CNS, central nervous system; AD, Alzheimer’s disease; PD, Parkinson’s disease; MS, multiple sclerosis; TLR4, toll-like receptor 4; NF-κB, nuclear transcription factor; HPLC, high-performance liquid chromatography; LOD, limit of detection; LOQ, limit of quantification; NOD, novel object discrimination test, TNF-α, tumor necrosis factor-α; IL-1β, interleukin-1β; NO, nitric oxide; iNOS, inducible nitric oxide synthase; CD11b/c, cluster differentiation 11b/c.

## References

[B1] Achoui M. Appleton D. Abdulla M. A. Awang K. Mohd M. A. Mustafa M. R. (2010). *In vitro* and *in vivo* anti-inflammatory activity of 17-*O*-acetylacuminolide through the inhibition of cytokines, NF-κB translocation and IKKβ activity. PLoS One 5, e15105. 10.1371/journal.pone.0015105 21152019 PMC2995738

[B2] Ambali S. F. Makinde A. O. Shittu M. Adeniyi S. A. Mowuogwu F. O. (2012). Alleviating effect of *Phyllanthus niruri* on sensorimotor and cognitive changes induced by subacute chlorpyrifos exposure in Wistar rats. Am. J. Med. Med. Sci. 2, 50–58. 10.5923/j.ajmms.20120203.05

[B3] Ashwlayan V. D. Singh R. (2011). Reversal effect of *Phyllanthus emblica* (Euphorbiaceae) Rasayana on memory deficits in mice. Int. J. App. Pharm. 3, 10–15.

[B4] Azmi N. Loh W. T. Omar S. S. Jalil J. Adam A. (2011). Effects of aqueous extract of *Prismatomeris glabra* root on non-spatial memory in rats using object discrimination test. Sains Malays 40, 1097–1103.

[B5] Brown R. E. Corey S. C. Moore A. K. (1999). Differences in measures of exploration and fear in MHC-congenic C57BL/6J and B6-H-2K mice. Behav. Genet. 29 (4), 263–271. 10.1023/A:1021694307672

[B6] Clausen F. Hanell A. Bjork M. Hillered L. Mir A. K. Gram H. (2009). Neutralization of interleukin-1β modifies the inflammatory response and improves histological and cognitive outcome following traumatic brain injury in mice. Eur. J. Neurosci. 30 (3), 385–396. 10.1111/j.1460-9568.2009.06820.x 19614750

[B7] Custódio C. S. Mello B. S. Cordeiro R. C. de Araújo F. Y. Chaves J. H. Vasconcelos S. M. (2013). Time course of the effects of lipopolysaccharide on prepulse inhibition and brain nitrite content in mice. Eur. J. Pharmacol. 713 (1–3), 31–38. 10.1016/j.ejphar.2013.04.040 23665499

[B8] Ennaceur A. (2010). One-trial object recognition in rats and mice: methodological and theoretical issues. Behav. Brain Res. 215, 244–254. 10.1016/j.bbr.2009.12.036 20060020

[B9] Farbood Y. Sarkaki A. Dianat M. Khodadadi A. Haddad M. K. Mashhadizadeh S. (2015). Ellagic acid prevents cognitive and hippocampal long-term potentiation deficits and brain inflammation in rat with traumatic brain injury. Life Sci. 124, 120–127. 10.1016/j.lfs.2015.01.013 25637685

[B10] Green L. C. Wagner D. A. Glogowski J. Skipper P. L. Wishnok J. S. Tannenbaum S. R. (1982). Analysis of nitrate, nitrite, and [15N] nitrate in biological fluids. Anal. Biochem. 126, 131–138. 10.1016/0003-2697(82)90118-X 7181105

[B11] Harikrishnan H. Jantan I. Haque M. A. Kumolosasi E. (2018). Phyllanthin from *Phyllanthus amarus* inhibits LPS-induced proinflammatory responses in U937 macrophages *via* downregulation of NF-κB/MAPK/PI3K-Akt signaling pathways. Phytother. Res. 32, 2510–2519. 10.1002/ptr.6190 30238535

[B12] Hernangomez M. Klusakova I. Joukal M. Hradilova-Svizenska I. Guaza C. Dubovy P. (2016). CD200R1 agonist attenuates glial activation, inflammatory reactions, and hypersensitivity immediately after its intrathecal application in a rat neuropathic pain model. J. Neuroinflammation 13, 43. 10.1186/s12974-016-0508-8 26891688 PMC4759712

[B13] Ilangkovan M. Jantan I. Mesaik M. A. Bukhari S. N. A. (2015). Immunosuppressive effects of the standardized extract of *Phyllanthus amarus* on cellular immune responses in Wistar–Kyoto rats. Drug Des. Devel. Ther. 9, 4917. 10.2147/DDDT.S88189 PMC4555964 26347462

[B14] Jantan I. Ilangkovan M. Mohamad H. F. (2014). Correlation between the major components of *Phyllanthus amarus* and *Phyllanthus urinaria* and their inhibitory effects on phagocytic activity of human neutrophils. BMC Complement. Altern. Med. 14, 429. 10.1186/1472-6882-14-429 PMC3659478 23737840

[B15] Joshi H. Parle M. (2006). Evaluation of antiamnestic potentials of [6]-gingerol and phyllanthin in mice. Nat. Prod. 2, 109–117.

[B16] Joshi H. Parle M. (2007). Pharmacological evidences for antiamnesic potentials of *Phyllanthus amarus* in mice. Afr. J. Biomed. Res. 10. 10.4314/ajbr.v10i2.50622

[B17] Kempuraj D. Thangavel R. Natteru P. A. Selvakumar G. P. Saeed D. Zahoor H. (2016). Neuroinflammation induces neurodegeneration. J. Neurol. Neurosurg. Spine 1, 1003. 28127589 PMC5260818

[B18] Kushwaha S. K. Dashora A. Dashora N. Patel J. R. Kori M. L. (2013). Acute oral toxicity studies of the standardized methanolic extract of *Phyllanthus amarus* Schum & Thonn. J. Pharm. Res. 67, 720–724. 10.1016/j.jopr.2013.04.020

[B19] Lawson-Evi P. Eklu-Gadegbeku K. Agbonon A. Aklikokou K. Moukha S. Creppy E. E. (2008). Toxicological assessment on extracts of *Phyllanthus amarus* Schum and Thonn. Sci. Res. Essays 39, 410–415.

[B20] Lima A. L. Alves A. F. Xavier A. L. Mozzini-Monteiro T. Oliveira T. R. Leite F. C. (2016). Anti-inflammatory activity and acute toxicity studies of hydroalcoholic extract of *Herissantia tiubae* . Rev. Bras. Farmacogn. 26, 225–232. 10.1016/j.bjp.2015.11.001

[B21] Liu G. Hu Y. Xiao J. Li X. Li Y. Tan H. (2016). 99mTc-labelled anti-CD11b SPECT/CT imaging allows detection of plaque destabilization tightly linked to inflammation. Sci. Rep. 6, 20900. 10.1038/srep20900 26877097 PMC4753504

[B22] Malve H. O. Raut S. B. Marathe P. A. Rege N. N. (2014). Effect of combination of *Phyllanthus emblica*, *Tinospora cordifolia*, and *Ocimum sanctum* on spatial learning and memory in rats. J. Ayurveda Integr. Med. 5, 209. 10.4103/0975-9476.146564 25624694 PMC4296432

[B23] Manalo R. V. Silvestre M. A. Barbosa A. L. A. Medina P. M. (2017). Coconut (*Cocos nucifera*) ethanolic leaf extract reduces amyloid-β (1-42) aggregation and paralysis prevalence in transgenic *Caenorhabditis elegans* independently of free radical scavenging and acetylcholinesterase inhibition. Biomedicines 5, 17. 10.3390/biomedicines5020017 PMC5489803 28536360

[B24] McDaniel K. L. Moser V. C. (1993). Utility of a neurobehavioral screening battery for differentiating the effects of two pyrethroids, permethrin and cypermethrin. Neurotoxicol. Teratol. 15 (2), 71–83. 10.1016/0892-0362(93)90065-V 8510610

[B25] Nillert N. Pannangrong W. Welbat J. U. Chaijaroonkhanarak W. Sripanidkulchai K. Sripanidkulchai B. (2017). Neuroprotective effects of aged garlic extract on cognitive dysfunction and neuroinflammation induced by β-amyloid in rats. Nutrients 9, 24. 10.3390/nu9010024 PMC5295068 28054940

[B26] Organisation for Economic Co-operation and Development (2002). Test no. 423: acute oral toxicity—acute toxic class method. Paris, France: OECD Publishing. 10.1787/9789264071001-en

[B27] Okun E. Barak B. Saada-Madar R. Rothman S. M. Griffioen K. J. Roberts N. (2012). Evidence for a developmental role for TLR4 in learning and memory. PLoS One 7, e47522. 10.1371/journal.pone.0047522 23071817 PMC3469493

[B28] Ownby R. L. (2010). Neuroinflammation and cognitive aging. Curr. Psychiatry Rep. 12 (1), 39–45. 10.1007/s11920-009-0082-1 20425309

[B29] Parajuli B. Sonobe Y. Kawanokuchi J. Doi Y. Noda M. Takeuchi H. (2012). GM-CSF increases LPS-induced production of proinflammatory mediators *via* upregulation of TLR4 and CD14 in murine microglia. J. Neuroinflammation 9 (1), 268. 10.1186/1742-2094-9-268 23234315 PMC3565988

[B30] Patel J. R. Tripathi P. Sharma V. Chauhan N. S. Dixit V. K. (2011). *Phyllanthus amarus*: ethnomedicinal uses, phytochemistry and pharmacology: a review. J. Ethnopharmacol. 138, 286–313. 10.1016/j.jep.2011.09.040 21982793

[B31] Radtke F. A. Chapman G. Hall J. Syed Y. A. (2017). Modulating neuroinflammation to treat neuropsychiatric disorders. BioMed Res. Int. 2017, 5071786. 10.1155/2017/5071786 29181395 PMC5664241

[B32] Rock R. B. Gekker G. Hu S. Sheng W. S. Cheeran M. Lokensgard J. R. (2004). Role of microglia in central nervous system infections. Clin. Microbiol. Rev. 17 (4), 942–964. 10.1128/CMR.17.4.942-964.2004 15489356 PMC523558

[B33] Shaftel S. S. Carlson T. J. Olschowka J. A. Kyrkanides S. Matousek S. B. O’Banion M. K. (2007). Chronic interleukin-1β expression in mouse brain leads to leukocyte infiltration and neutrophil-independent blood–brain barrier permeability without overt neurodegeneration. J. Neurosci. 27, 9301–9309. 10.1523/JNEUROSCI.1418-07.2007 17728444 PMC6673122

[B34] Shen Y. McMackin M. Z. Shan Y. Raetz A. David S. Cortopassi G. (2016). Frataxin deficiency promotes excess microglial DNA damage and inflammation that is rescued by PJ34. PLoS One 11, e0151026. 10.1371/journal.pone.0151026 26954031 PMC4783034

[B35] Strużynska L. Dąbrowska-Bouta B. Koza K. Sulkowski G. (2006). Inflammation-like glial response in lead-exposed immature rat brain. Toxicol. Sci. 95, 156–162. 10.1093/toxsci/kfl134 17047031

[B36] Thomson L. M. Sutherland R. J. (2005). Systemic administration of lipopolysaccharide and interleukin-1β have different effects on memory con­solidation. Brain Res. Bull. 67, 24–29. 10.1016/j.brainresbull.2005.05.024 16140159

[B37] Tong F. Zhang J. Liu L. Gao X. Cai Q. Wei C. (2016). Corilagin attenuates radiation-induced brain injury in mice. Mol. Neurobiol. 53, 6982–6996. 10.1007/s12035-015-9591-6 26666668

[B38] Tortorelli L. S. Engelke D. S. Lunardi P. Mello e Souza T. Santos-Junior J. G. Gonçalves C. A. (2015). Cocaine counteracts LPS-induced hypolocomotion and triggers locomotor sensitization expression. Behav. Brain Res. 287, 226–229. 10.1016/j.bbr.2015.03.054 25835320

[B39] Uddin M. S. Mamun A. A. Iqbal M. A. Islam A. Hossain M. F. Khanum S. (2016). Analyzing nootropic effect of *Phyllanthus reticulatus* Poir. Adv. Alzheimer. Dis. 5, 87–102. 10.4236/aad.2016.53007

[B40] Yuandani I. J. Ilangkovan M. Husain K. Chan K. M. (2016). Inhibitory effects of compounds from *Phyllanthus amarus* on nitric oxide production, lymphocyte proliferation, and cytokine release from phagocytes. Drug Des. Devel. Ther. 10, 1935. 10.2147/DDDT.S105651 PMC4907639 27354767

[B41] Zarifkar A. Choopani S. Ghasemi R. Naghdi N. Maghsoudi A. H. Maghsoudi N. (2010). Agmatine prevents LPS-induced spatial memory impairment and hippocampal apoptosis. Eur. J. Pharmacol. 634, 1–3, 84–88. 10.1016/j.ejphar.2010.02.029 20184876

[B42] Zhang Y. Xu T. Pan Z. Ge X. Sun C. Lu C. (2018). Shikonin inhibits myeloid differentiation protein 2 to prevent LPS-induced acute lung injury. Br. J. Pharmacol. 175, 840–854. 10.1111/bph.14129 29243243 PMC5811619

[B43] Zhao X. Liao Y. Morgan S. Mathur R. Feustel P. Mazurkiewicz J. (2018). Noninflammatory changes of microglia are sufficient to cause epilepsy. Cell Rep. 22, 2080–2093. 10.1016/j.celrep.2018.02.004 29466735 PMC5880308

[B44] Zhu L. Nang C. Luo F. Pan H. Zhang K. Liu J. (2016). Esculetin attenuates lipopolysaccharide (LPS)-induced neuroinflammatory processes and depressive-like behavior in mice. Physiol. Behav. 163, 184–192. 10.1016/j.physbeh.2016.04.051 27133730

