# Supplementary figures and images for: Protective Effects of Phyllanthus amarus Against Lipopolysaccharide-Induced Neuroinflammation and Cognitive Impairment in Rats
Source: Front Pharmacol. 2019 Jun 4;10:632. doi: 10.3389/fphar.2019.00632 (PMC6558432; doi:10.3389/fphar.2019.00632)

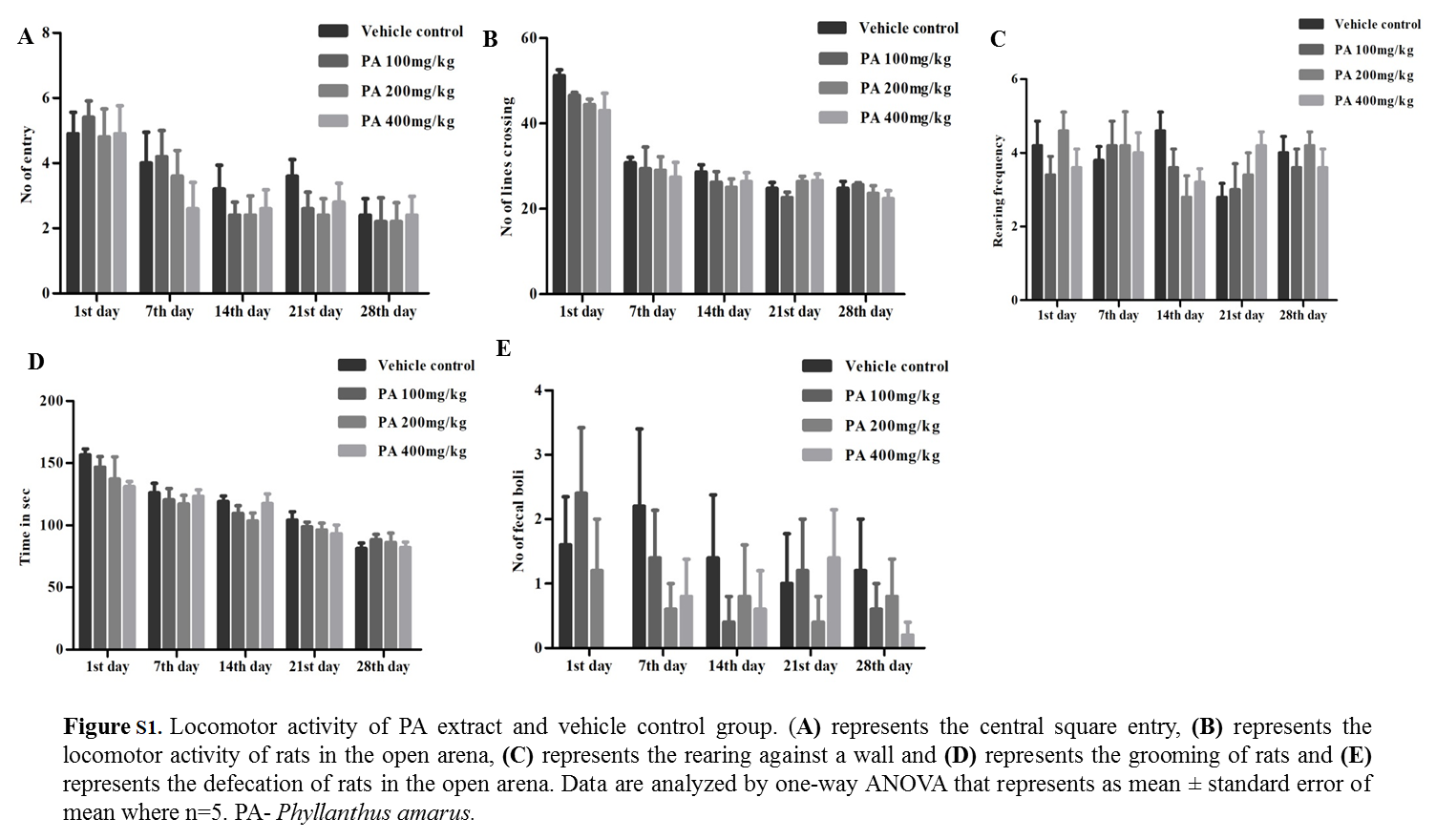

Supplement: Supplementary file 1 [file Image_1.tif]

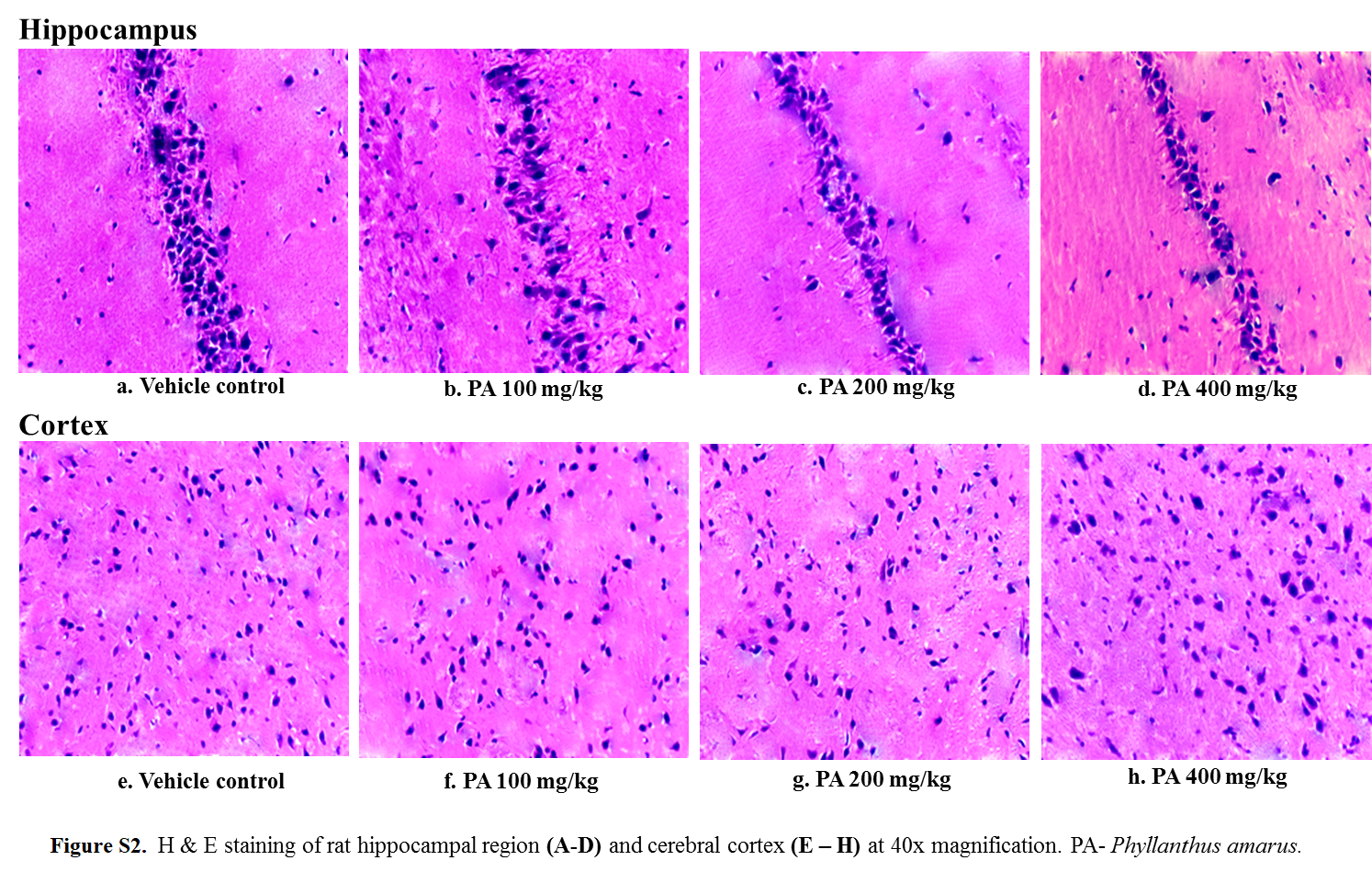

Supplement: Supplementary file 2 [file Image_2.tif]
